# Diagnostic Accuracy of Mid-Upper Arm Circumference for the Detection of Acute Malnutrition Among Children Aged 6–60 Months: A Diagnostic Accuracy Study

**DOI:** 10.34172/jrhs.2024.147

**Published:** 2024-06-01

**Authors:** Krishna M. Jasani, Vibha V. Gosalia, Shobha V. Misra

**Affiliations:** ^1^All India Institute of Medical Sciences, Rajkot, Gujarat, India; ^2^Department of Community Medicine, PDU Government Medical College, Rajkot, India

**Keywords:** Child malnutrition, Receiver operating characteristics, Area under curve, World Health Organization

## Abstract

**Background:** Timely and accurate screening of malnutrition at the community level is essential to identifying malnourished children. The World Health Organization (WHO) guidelines classify non-oedematous acute malnutrition among children using mid-upper arm circumference (MUAC) or weight-for-height Z-score (WHZ).

**Study Design:** A cross-sectional study.

**Methods:** This study was conducted among children aged 6‒60 months. After necessary exclusions, 433 participants were selected using a multi-stage simple random sampling method. Using WHO guidelines for global acute malnutrition (GAM) [WHZ<-2, MUAC<12.5 cm], the sensitivity (Se), specificity (Sp), predictive values, likelihood ratios, Youden index, and receiver operating characteristic (ROC) curve were calculated for MUAC using WHZ as the criterion.

**Results:** Out of 433 participants, 30% were diagnosed with GAM using WHZ, while 17.6% were found malnourished using MUAC measurements. As per WHO cut-offs, the Se, Sp, positive predictive value (PPV), negative predictive value (NPV), Youden index, positive likelihood ratio (LR+), and negative likelihood ratio (LR-) of MUAC were 48%, 96%, 83%, 81%, 0.44, 12, and 0.54, respectively. The ROC curve displayed an area under the curve of 0.86 (95% confidence interval=0.83, 0.90) for MUAC<12.5 cm. Bivariate Pearson correlation also demonstrated a positive linear relationship (R2=0.302) between the WHZ and MUAC variables.

**Conclusion:** Based on the findings, 48% of the children were correctly identified by the MUAC with an 83% probability of GAM (PPV=0.83). Moreover, there was 96% Sp in non-malnourished children, with only 4% false positives. Therefore, personnel at the grassroots level can use MUAC for timely and accurate screening of children in Anganwadi centers (AWCs) due to its ease of use and simplicity.

## Background

 Acute malnutrition continues to be a major public health concern worldwide. In 2022, globally, 45.0 million children under five were wasted, of which 13.7 million were severely wasted. This translates into a prevalence of 6.8% and 2.1%, respectively.^1^ These children are vulnerable to short-term risks of disease, impaired development, and mortality, as well as irreversible long-term risks such as short stature and impaired cognition.^2^ Undernutrition is estimated to be associated with 45% of the deaths among children under five every year, occurring mostly in low- and middle-income countries. Moreover, a severely wasted child has a more than 11-fold increased risk of death compared to a non-wasted child.^3^ In 2019‒2020, India had 19.3% wasted children, with 7.7% severely wasted, as per the National Family Health Survey–5 (NFHS) report. This prevalence was found to be greater in rural areas as compared to urban areas in India.^4^ In countries with a high prevalence of malnutrition, timely and accurate screening at the community level is essential to identify children with acute malnutrition; accordingly, 500 000 deaths per year could be prevented globally by timely and proper treatment of acute malnutrition.^5^

 Accurate recording of weight and height/length and their subsequent translation into weight-for-height/length Z-scores can be quite challenging in community settings for health workers. Several large community management of acute malnutrition programs are therefore increasingly utilizing mid-upper arm circumference (MUAC) to identify global acute malnutrition (GAM). MUAC is a widely used tool, especially in resource-limited countries, to identify waste. This measurement offers advantages because it requires minimal equipment and is simple, relatively inexpensive, and reasonably accurate.^6^

 The present study was conducted in the Rajkot district, the fourth largest city in Gujarat state of India, where the wasting prevalence was 17.6% according to the District Level Household Survey-5 (DLHS-5),^7^ which is on the decline (23.4% in DLHS-4).^8^ Despite improvements in acute malnutrition prevalence, the level of severe wasting increased dramatically in 2019–20. (3.7% in DLHS-4 to 7.3% in DLHS-5). To measure non-oedematous acute malnutrition in children aged 6–60 months, the World Health Organization (WHO) guidelines endorse the use of MUAC or weight-for-height Z-score (WHZ) to classify severe acute malnutrition (WHZ < -3, MUAC < 11.5 cm), moderate acute malnutrition (WHZ -3 to < -2, MUAC 11.5 to < 12.5 cm), and GAM (WHZ < -2, MUAC < 12.5 cm).^9,10^ Considering that WHZ and MUAC are the major anthropometric tools used to identify wasting in children, comparing the accuracy of both tools to identify wasting provides a better sense of the measurements. In the AWCs of the Rajkot district for the identification of malnourished children, Anganwadi workers plot the weight of the child for age in the growth chart of a particular child and identify the child with a low weight for age. Thus, MUAC or WHZ has not been used in the AWCs. Hence, by evaluating the diagnostic accuracy of these two tools, any one tool can be used for screening and identification of acute malnutrition in primary healthcare (PHC) settings.

 Therefore, the aim of the current study is to determine the diagnostic accuracy of MUAC versus WHZ in the detection of acute malnutrition in the rural children of the Rajkot district.

## Methods

 Between March 2021 and May 2022, an analytic cross-sectional study was conducted among children up to six years of age registered in the Anganwadi centers (AWCs) of the Rajkot district in western India.

 Based on DLHS-5 data of Rajkot District,^7^ the prevalence of wasting is 17.6%. To obtain the sample size, the formula N = Z^2^pq/e^2^ was applied, and 20% of the relative sample error was taken into account. Accordingly, the sample yield for the prevalence of wasting was 450. The final sample size of 504 was calculated after accounting for 10% of non-respondents. A multi-stage sampling method was utilized to achieve the desired sample size of 504.

 Initially, 14 PHC centers within 35 km of PDU Government Medical College were selected based on feasibility criteria from the 54 in the Rajkot district.

 The second step involves enlisting all AWCs from each selected PHC center in alphabetical order and choosing three AWCs by the lottery method.

 As a third step, from each of the selected AWCs, a list of all children aged 0–6 years was obtained, and 12 children were selected from that list by means of simple random sampling.

 It was decided that the next eligible child would be taken in the study if the selected child was absent or their caretakers did not give their consent.

 As per the WHO’s criteria, MUAC was measured in children aged 6‒60 months. Therefore, this study excluded children aged 0‒6 months and 5‒6 years. Furthermore, children with clinical signs of oedema (which can increase body weight and compromise WHZ and MUAC reliability), children who failed to thrive, had congenital defects, or whose parents/caregivers could not give consent, were excluded from the investigation. Thus, the final sample size was 433 after necessary exclusion.

 Prior to enrolling any study subjects, the Institutional Ethical Committee (Human) authorized the final study protocol, along with the questionnaire and consent forms.

###  Measures

 Eligible children, after informed consent was given, had anthropometrics measured (weight, height/length, and MUAC) by the researchers according to a standardized protocol and the use of calibrated equipment.

 Using a Salter’s scale (pre-calibrated or electric), the child’s weight was calculated while wearing just light clothing and without shoes, rounding up to the nearest 0.5 kg. An infantometer and stadiometer were used to calculate length and height for children < 2 years and > 2 years, respectively, with a 0.5 cm tolerance for errors. MUAC was measured using Shakir’s tape. To locate the midpoint of the left arm, it was flexed at the right angle, and the midpoint was marked between the acromion and olecranon processes. The arm was allowed to hang freely. Then, color-coded, non-stretchable Shakir’s tape was placed snugly around the arm at the midpoint mark, and measurements were noted to the nearest 1 mm for accuracy.

###  Variables

 By integrating the WHO 2006 multi-center growth reference data into the computer program WHO Anthro 2011 (version 3.2), wasting was evaluated using the anthropometric index WHZ, stunting with L/HAZ and underweight with WAZ. Malnutrition was determined to be a z-score of < -2 SD below the median value of the reference population. It was classified as moderate when the Z-score was between -2 and -3 SD and as severe when the Z-score was < -3 SD. MUAC measurements under the red category with < 11.5 cm were considered severe, while measurements between 11.5 cm and 12.5 cm were considered moderate under the yellow category. To measure non-oedematous acute malnutrition in children aged 6–60 months, both MUAC and WHZ indicators were dichotomized according to the WHO guidelines for GAM (WHZ < -2 and MUAC < 12.5 cm).

###  Data entry and analysis

 After being verified to be accurate, the gathered information was imported into Microsoft Office Excel 2013. Epi-Info software (Version 7.1.5) from the CDC, Atlanta, United States, was used for the analysis. Means and standard deviations were calculated for height, weight, and MUAC. The frequencies of the categorized standard WHO growth indicators were calculated (WHZ, WAZ, L/HAZ, and MUAC). For descriptive purposes, these variables were stratified by age and gender, and a test of significance (the chi-square test) was applied to check the association between variables. An independent t-test was performed to compare the means of anthropometric measures across various subgroups. The results with a *P*-value < 0.05 at a 95% confidence interval (CI) were considered to be statistically significant. Contingency tables were prepared to calculate sensitivity (Se), specificity (Sp), positive predictive values (PPV), and negative predictive values (NPV), which were stratified by dichotomous variables (gender and age groups).

 Likelihood ratios (LR + and LR-) were calculated to assess the effect of the MUAC measurement on the probability of malnutrition. The Youden index was estimated according to the formula (Se + Sp) – 1 for the goodness of the test. Resultant values ranged between 0 and 1, with 0 and 1 indicating no differential ability and complete differential ability, respectively, giving equal weight to Se and Sp.

 Receiver operating characteristic (ROC) analysis and area under the curve (AUC) index were used to assess the performance of MUAC as a diagnostic test when using WHZ as the criterion for acute malnutrition. The ROC analysis plots the Se and 1-Sp of the diagnostic test (MUAC) against the diagnostic criteria WHZ for the diagnosis of GAM. The AUC obtained from the ROC analysis ranges between 0 and 1, where 0.5 and 1 indicate an ineffective test (50% accuracy) and a perfect test (100% accuracy), respectively. The upper and lower 95% CI of the AUC were provided as well. An adjusted ROC analysis was conducted to obtain the AUC for distinct subgroups categorized by age and gender. This is aimed at assessing the discriminatory performance of the MUAC across various demographic segments, allowing for a nuanced understanding of predictive efficacy within specific age and gender cohorts. The regression ROC (r-ROC) curve was also plotted to evaluate the predictive performance of the logistic regression model. It assessed the ability of a regression model to predict the probability or likelihood of MUAC < 12.5 cm, typically in the context of identifying GAM categorized by age group and gender.

## Results

###  Sample characteristics

 A sample of 433 children aged 6–60 months was included in the analysis. The median age of the children was 31 (inter-quartile range = 26) months, with 36.7% aged 6–24 months and 63.3% aged 25–60 months. The male-to-female ratio was almost equal in our study. The mean weight, height, and MUAC of the children were 11.3 ± 2.9 kg, 87.4 ± 12.3 cm, and 13.9 ± 1.6 cm, respectively. The t-test comparing weight between males and females demonstrated a *P* value of 0.04, indicating a significant difference in weight between the genders. Conversely, for height and MUAC, no statistically significant differences were observed between males and females (*P*= 0.111 and 0.462, respectively), suggesting that these anthropometric measures do not significantly differ between the genders. Additionally, when comparing mean weight and mean height between different age groups, statistically significant differences were found for both variables (*P*< 0.001), implying substantial differences in both weight and height across the specified age groups.

###  Malnutrition prevalence estimation

 Anthropometric indicators (WHZ, WAZ, HAZ, and MUAC) categorized by current WHO classifications are presented in Table 1. Nearly one-third (30%) of the children were wasted (WHZ < -3 = 7.9%, WHZ 3 to < -2 = 22.2%). Severe wasting was found more in males as compared to females, and this difference was statistically significant (*P*= 0.009). More than one-third (37.2%) were found stunted (HAZ < -3 = 10.4%, HAZ 3 to < -2 = 26.8%). Underweight was found among 40.4% of children (WAZ < -3 = 7.4%, HAZ 3 to < -2 = 33%). Children aged between 24 and 60 months exhibited a higher prevalence of moderate underweight compared to those aged between 6 and 24 months, and this difference was found to be statistically significant (*P*= 0.032). Nearly one-fifth (17.6%) of children had MUAC < 12.5 cm of whom, 3.9% fell into the red category of MUAC measurement. A total of 76 (17.6%) children were identified as GAM by MUAC measurement, as compared to 130 (30%) by WHZ.

**Table 1:** Descriptive summary of anthropometric measures and indicators, stratified by gender and age (N = 433)

**Variables**	**Male (n=217)**		**Female (n=216)**		***P*** ** value**	**Age 6-24 (mon), n=159**		**Age 24-60 (mon), n=274**		***P*** ** value**
**Anthropometric Measures**	**Mean**	**SD**	**Mean**	**SD**		**Mean**	**SD**	**Mean**	**SD**	
Weight (kg)	11.5	2.8	11	3.1	0.042	8.8	1.6	12.7	2.5	0.001
Height (cm)	88.4	11.7	86.5	12.7	0.111	75.3	6.8	94.5	8.7	0.001
MUAC (cm)	13.4	2.0	14	1.5	0.463	13.8	1.4	13.9	1.6	0.601
**Anthropometric indicators**	**No.**	**%**	**No.**	**%**	***P *** **value**	**No.**	**%**	**No.**	**%**	***P *** **value**
Weight for height Z-score										0.590
< -3^a^	25	11.5	9	4.2	0.009	10	6.3	24	8.8	
-3 to < -2^b^	46	21.2	50	23.1		33	20.8	63	23	
Weight for age Z-score										0.031
< -3^c^	14	6.5	18	8.3	0.161	15	9.4	17	6.2	
-3 to < -2^d^	82	37.8	61	28.2		40	25.2	103	37.6	
Height for age Z-score										0.482
< -3^e^	24	11.1	21	9.7	0.854	19	11.9	26	9.5	
-3 to < -2^f^	60	27.6	56	25.9		56	35.2	60	21.9	
MUAC (cm)										0.522
< 11.5^a^	12	5.5	5	2.3	0.222	4	2.5	13	4.7	
11.5 to < 12.5^b^	32	14.7	27	12.5		21	13.2	38	13.9	

*Note*. SD: Standard deviation; MUAC: Mid upper arm circumference. ^a^Severe acute malnutrition; ^b^Moderate acute malnutrition;^c^Severe underweight; ^d^Moderate underweight; ^e^Severe stunting; ^f^Moderate stunting.

###  Diagnostic performance of mid-upper arm circumference

**Figure 1:**
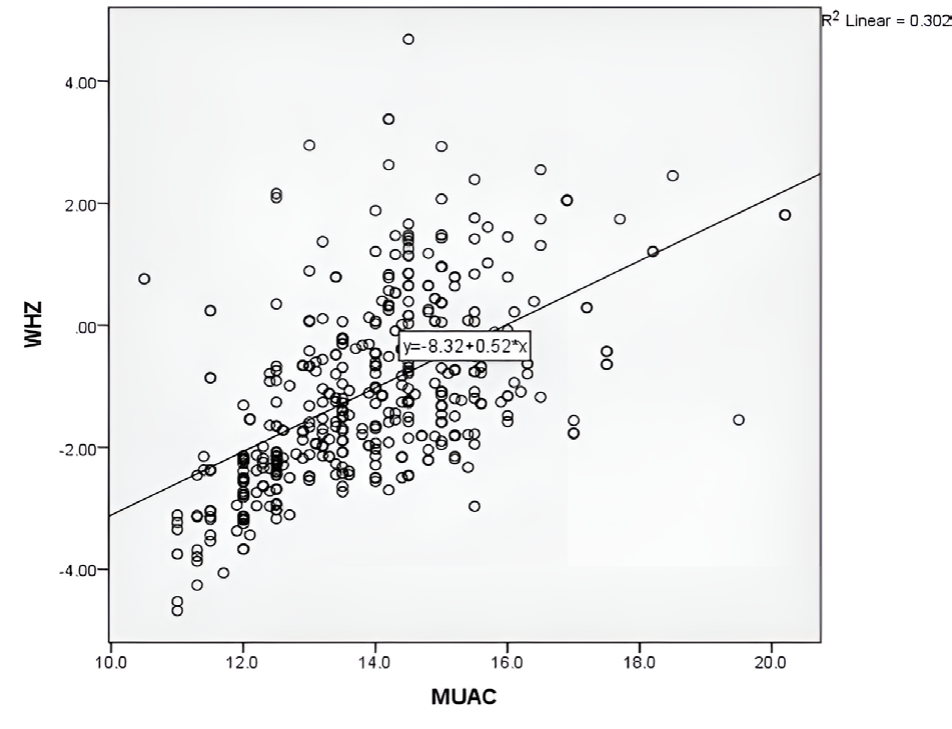
Pearson correlation of global acute malnutrition as determined by weight-for-height Z-scores (Y-axis) with mid-upper arm circumference (X-axis) in given samples

Figure 1 shows the bivariate Pearson correlation of WHZ and MUAC variables, which demonstrates a positive linear relationship (R^2^ = 0.302). It suggests that as WHZ increases, MUAC tends to increase as well, and as WHZ decreases, MUAC tends to decrease, which could be important for monitoring the nutritional status of the children. The *P* value for the correlation between WHZ and MUAC was also found to be statistically significant (*P*< 0.001).

 When MUAC < 12.5 cm was considered to identify GAM when using WHZ as a criterion according to WHO cut-offs (WHZ < -2 present or WHZ < -2 not present) in the total sample for children stratified by age and gender (Table 2), the total Se of MUAC < 12.5 cm was 0.48, which showed that 48% of the children had both MUAC < 12.5 cm and WHZ < -2 (indicating malnutrition), which were correctly identified by the MUAC measurement. The Se of MUAC < 12.5 cm was 0.96, indicating that 96% of children had both MUAC 12.5 cm and WHZ > -2 (indicating no malnutrition), which were correctly identified by the MUAC measurement as negative. It correctly identified 96% of non-malnourished children, with only a 4% false-positive rate. Among those who had a positive GAM screening test (MUAC < 12.5 cm), the probability of GAM was 83% (PPV = 0.83) according to the WHZ < -2 criterion, whereas the NPV of 0.81 suggested that there was an 81% probability that children with a negative MUAC test result were indeed free from GAM according to the WHZ criteria. A LR + of 12 suggested that MUAC < 12.5 cm was associated with a 12-fold increase in the likelihood of having GAM, while a LR− of 0.54 represented that the negative test result (MUAC ≥ 12.5 cm) was associated with a 46% reduction in the likelihood of having GAM. A Youden index of 0.44 demonstrated that the MUAC screening test, with a cut-off of 12.5 cm, had relatively good discriminative ability in distinguishing between children with and without GAM. Based on the data in Table 2, the Se and Sp of MUAC measurements were high in the age group of 24‒60 months and male children.

**Table 2:** Measures of diagnostic performance for MUAC < 12.5 cm to identify global acute malnutrition, stratified by age and gender*

**Variables**	**Sensitivity**	**Specificity**	**PPV**	**NPV**	**LR+**	**LR−**	**Youden Index**	**AUC (95% CI)**
Total	0.48	0.96	0.83	0.81	12.0	0.54	0.44	0.865 (0.83, 0.90)
Age (mon)								
6‒24	0.44	0.95	0.76	0.82	8.8	0.59	0.39	0.847 (0.77, 0.92)
25‒60 m	0.51	0.96	0.86	0.81	12.7	0.51	0.47	0.872 (0.83, 0.93)
Gender								
Male	0.55	0.97	0.89	0.82	18.3	0.46	0.52	0.854 (0.80, 0.92)
Female	0.41	0.95	0.75	0.81	8.2	0.62	0.36	0.875 (0.83, 0.92)

*Note*. CI: Confidence interval; MUAC: MUAC: Mid upper arm circumference; ROC: Receiver operating characteristic; WHO: World Health Organization; WHZ: Weight-for-Height Z-score; GAM: Global acute malnutrition; PPV: Positive predictive value; NPV: Negative predictive value; LR + : Positive likelihood ratio; LR: Negative likelihood ratio; AUC: Area under curve. ^*^Contingency tables and ROC analysis were used to assess the diagnostic performance of MUAC < 12.5 cm to identify GAM when using WHZ as a criterion according to WHO cut-offs (present WHZ < -2 or not present WHZ < -2) in the total sample for children (N = 433) and stratified by age and gender.

**Figure 2:**
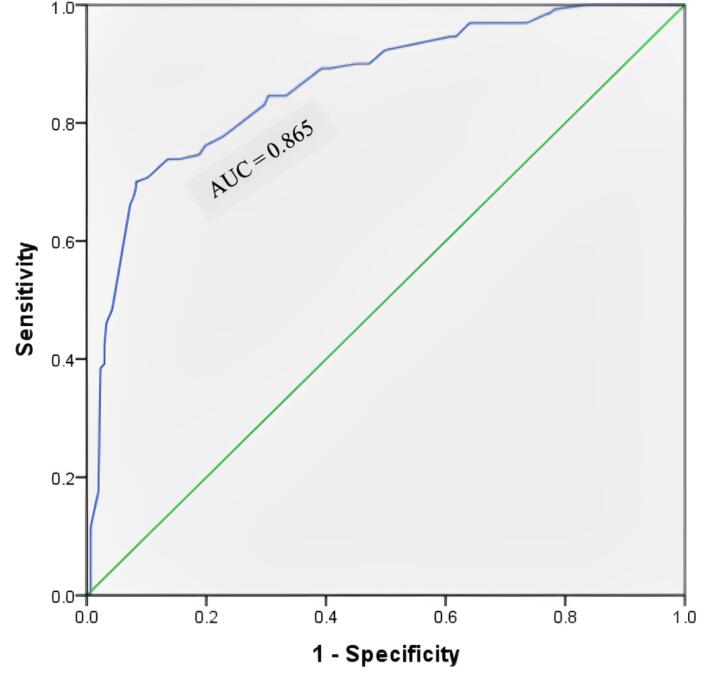
Receiver operating characteristic curve for the diagnosis of global acute malnutrition using mid-upper arm circumference in the age group of 6‒60 months (N = 433). *Note*. The receiver operating characteristic curve shows the ability of mid-upper arm circumference to diagnose children with global acute malnutrition in the age group of 6-60 months [area under curve = 0.865 (95% confidence interval = 0.83‒0.90)]

**Figure 3:**
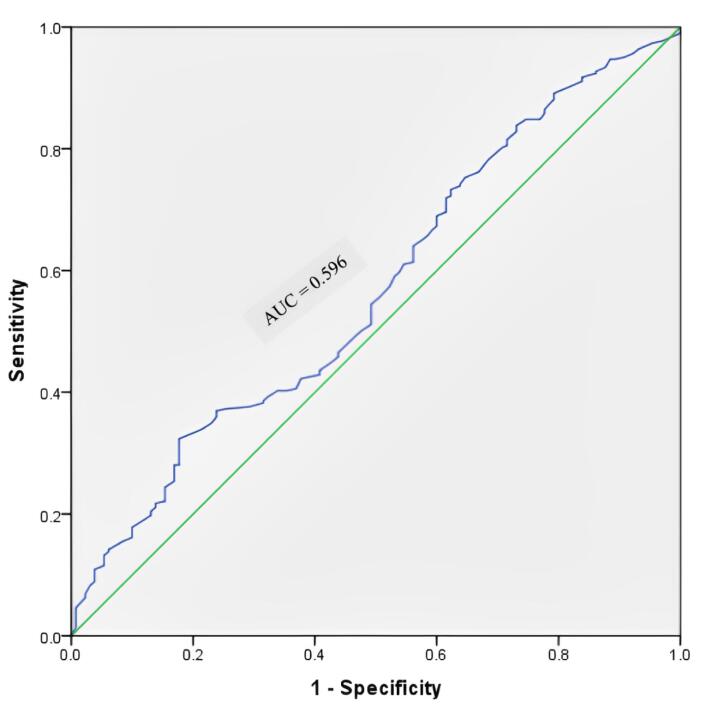
Regression receiver operating characteristic curve for the diagnosis of global acute malnutrition using mid-upper arm circumference in the age group of 6‒60 months (N = 433). *Note*. The regression receiver operating characteristic curve illustrates the ability of mid-upper arm circumference to diagnose children with global acute malnutrition in the age group of 6‒60 months [area under curve = 0.596 (95% confidence interval = 0.51‒0.63)]

Figure 2 illustrates the ROC curve of MUAC when using WHZ as the criterion. As shown, the MUAC-ROC curve is above the WHZ curve. The ROC curve displays an AUC of 0.865 (95% CI = 0.83‒0.90) with 48% Se and 96% Sp for MUAC < 12.5 cm in the intended samples. The adjusted ROC analysis revealed that female children exhibit a higher AUC in relation to MUAC < 12.5 cm compared to male children. Additionally, children aged 25‒60 months demonstrated a superior AUC relative to those aged 6‒24 months. These findings suggest that MUAC serves as a more discerning predictor for the outcome in female children and older age groups (25‒60 months) in comparison to their respective counterparts (Table 2). The regression ROC (r-ROC) analysis yielded an AUC value of 0.569, with a 95% CI ranging from 0.51 to 0.63, after eliminating potential confounding factors. It suggests that the model has some discriminative ability in predicting the outcome, performing slightly better than chance. The asymptotic significance level associated with the AUC value was 0.023, indicating the statistical significance of the AUC estimate, as this is < 0.05 (Figure 3).

## Discussion

 Our findings demonstrated a 30.5% wasting prevalence, which was in accordance with the results of research performed in Assam^11^ and higher than the estimations of the national, state, and regional averages reported in the NFHS–5^4^ (19.3%, 25.1%, and 17.6%, respectively). Additionally, this prevalence was higher in comparison to the results of earlier studies.^12-15^ The findings of the current study revealed an 8% prevalence of severe wasting, which is consistent with the results of the study conducted in Gujarat (6%),^16^ Haryana (8%),^13^ and with the national average (7.7%).^4^ In the current study, the prevalence of stunting (36.7%) was slightly lower than it was for the state (39%) and the region (38.9%). The prevalence of underweight in participants (39.1%) was similar to that of the state (39.7%) and higher than the regional average (37%). In the present study, 17.6% of participants were found malnourished by MUAC measurements, which was almost doubled in a study performed by Abitew et al.^17^

 The advantage of MUAC measurements over WHZ criteria in diagnosing GAM is their simple, rapid tool and their great advantages over the operational problem of taking height and weight in resource-poor settings. Based on the results of the current study, a significant positive linear correlation was found between MUAC and WHZ (Pearson correlation: 0.302,* P* < 0.001) to detect GAM, which was comparable to the results of studies conducted in Kolkata^18^ and Bangladesh.

 This study reported a Se of 48% at the WHO-suggested MUAC cut-off of < 12.5 cm, which was almost similar to the results of a study performed in rural Uttar Pradesh^6^. There was a large range in the Se of MUAC, ranging between 16.6% and 79% in various studies.^2,5,20-23^

 In the present study, the Sp of the MUAC cut-off of 12.5 cm was 96%, which was consistent with the findings of Mane et al,^21^ Sougaijam et al,^22^ and Sendaula et al.^23^ Compared to our study, the Sp reported in other earlier studies^2,3,20,24,25^ was lower than our findings.Whereas, Laillou et al^5^ and Talapalliwar and Garg^26^ reported specificities of 97% and 98.2%, respectively, which were slightly higher than our findings.

 Based on the findings, the AUC of 0.865 (95% CI = 0.83‒0.90) was comparable to the results of Sendaula et al^23^ and Marshall et al,^2^ while the other two previous studies^5,25^ reported a lower AUC than ours. The PPV of GAM and NPV were 83% and 81% in the current study, which contradicts the results of the study conducted in Kolkata,^18^ Madhya Pradesh,^20^ Karnataka,^25^ and Uganda.^23^ Our results represented a Youden index of 0.44, which was lower than that of the study by Sougaijam et al^22^ and higher than the study performed in Maharashtra^26^ and Nepal.^3^ According to the LR + of 12, MUAC < 12.5 was associated with a 12-fold increased risk of GAM. This was higher than the results of studies conducted by Shukla et al^20^ and Ranadip et al.^18^ However, the LR− of 0.54 indicates that MUAC ≥ 12.5 cm significantly reduced GAM risk by 46%, which is consistent with the results of other studies.^18,20^

**Highlights**

- MUAC demonstrates moderate sensitivity and high specificity in detecting acute malnutrition.
- There is a positive correlation between MUAC and WHZ measurements.
- MUAC < 12.5 cm shows a strong predictive ability for acute malnutrition.

## Conclusion

 The prevalence of malnutrition by WHZ is higher than that by MUAC in the present study. At the community level, the primary aim is to comprehensively identify all cases of acute malnutrition. In our study, 48% of the children were correctly identified by the MUAC measurement with an 83% probability of GAM (PPV = 0.83) and 96% Sp in non-malnourished children, with only 4% false positives.Accordingly, a combined approach of MUAC and WHZ should be advocated for screening and diagnosis purposes in a community setting, given its ease of use. The findings of this study indicated that MUAC < 12.5 cm was linked to a 12-fold increase in the chance of developing GAM, with an LR + of 12. However, in contexts prioritizing the management of malnourished children, the utilization of MUAC alone demonstrated a marked reduction in caseload by approximately 96%, specifically targeting cases necessitating urgent intervention for GAM. Thus, these findings enhance the evidence base to support the use of MUAC as a screening tool for early detection, prompt, and appropriate treatment of acute malnutrition.

## Limitations of the Study

 The study was only performed among Anganwadi registered children. Thus, children who were not beneficiaries of Anganwadis were not a part of this study.

## Acknowledgments

 The authors acknowledge all the participants who gave consent and participated in the study.

## Authors’ Contribution

**Conceptualization:** Krishna M. Jasani, Vibha V. Gosalia, Shobha V. Misra.

**Data curation:** Krishna M. Jasani.

**Formal analysis:** Krishna M. Jasani.

**Investigation:** Krishna M. Jasani.

**Methodology:** Krishna M. Jasani, Vibha V. Gosalia, Shobha V. Misra.

**Project administration:** Vibha V. Gosalia, Shobha V. Misra.

**Software:** Krishna M. Jasani.

**Supervision:** Vibha V. Gosalia, Shobha V. Misra.

**Validation:** Vibha V. Gosalia, Shobha V. Misra.

**Visualization:** Vibha V. Gosalia, Shobha V. Misra.

**Writing–original draft:** Krishna M. Jasani.

**Writing–review & editing:** Krishna M. Jasani, Vibha V. Gosalia.

## Competing Interests

 The authors declare no conflict of interests.

## Ethical Approval

 The final study protocol, including the final version of the questionnaire and consent forms, was approved by the Institutional Ethical Committee (Human), PDU Medical College Rajkot (PDUMCR/IEC/162/2021) before the enrolment of any study subject in the study.

## Funding

 The study received no funding sources.
